# Combined Characterization of the Time Response of Impression Materials via Traditional and FTIR Measurements

**DOI:** 10.3390/ma8052387

**Published:** 2015-05-06

**Authors:** Giacomo Derchi, Enrico Manca, Amirreza Shayganpour, Antonio Barone, Alberto Diaspro, Marco Salerno

**Affiliations:** 1 Department of Surgical, Medical, Molecular and Critical-Area Pathology, University of Pisa, via Paradisa 2, I-56124 Pisa, Italy; E-Mails: gnolo78@gmail.com (G.D.); barosurg@gmail.com (A.B.); 2 Tuscan Stomatological Institute, via Aurelia 335, Lido di Camaiore, I-55041 Lucca, Italy; E-Mail: info@enricomanca.it; 3 Nanophysics Department, Istituto Italiano di Tecnologia, via Morego 30, I-16163 Genova, Italy; E-Mails: amirreza.shayganpour@iit.it (A.S.); alberto.diaspro@iit.it (A.D.); 4 University of Genova, viale Causa 13, I-16145 Genova, Italy

**Keywords:** dental impression materials, polyether, siloxane, working time, setting time, Shore hardness, shark fin test, Fourier‑transform infrared spectroscopy

## Abstract

We investigated the temporal response of four dental impression materials, namely three siloxanes (Imprint 4, Flexitime, Aquasil) and one polyether (Impregum). The null hypothesis was that the nominal working times are confirmed by instrumental laboratory tests. We also aimed to identify alternative techniques with strong physical-chemical background for the assessment of temporal response. Traditional characterization was carried out by shark fin test device and durometer at both ambient and body temperature. Additionally, Fourier-transform infrared spectroscopy was performed at room temperature. From shark fin height and Shore hardness *versus* time the working time and the setting time of the materials were evaluated, respectively. These were in reasonable agreement with the nominal values, except for Impregum, which showed longer working time. Spectroscopy confirmed the different character of the two types of materials, and provided for Imprint 4 and Aquasil an independent evaluation of both evolution times, consistent with the results of the other techniques. Shark fin test and durometer measurements showed deviations in setting time, low sensitivity to temperature for Flexitime, and longer working time at higher temperature for Impregum. Deviations of working time appear in operating conditions from what specified by the manufacturers. Fourier-transform infrared spectroscopy can provide insight in the correlation between material properties and their composition and structure.

## 1. Introduction

Today several types of dental impression materials exist, including polysulphides, based on traditional covalent bonding between polymer networks, and alternative polymers with networking based on Hydrogen bonding, for example the two polysaccharides of agar and alginate [1]. However, the most common impression materials used are still polyethers (PEs) and addition silicones (ASs) [2]. The PEs are based on moderately low molecular weight PE, silica filler and plasticizer, which on mixing give rise to a rubber by cationic polymerization. ASs are based on a main chain containing Silicon (Si), geometrically similar to the Carbon (C) chain of resins. Materials from both classes may exhibit excellent dimensional stability and wettability, which means minimal voids [1,2,3].

The major operating parameters of impression materials correlate with the time elapsed from mixing the two components that start the reticulation. The working time *t*_w_ can be defined as the time during which there is no significant change in the rheological and mechanical properties of the material, such that re-arrangement of the material is still possible within this time; the setting time *t*_s_ is the time for the network of reticulated polymer to take place, converting the viscous liquid to a stable stretch-resistant solid. While *t*_w_ < *t*_s_ by definition, the ideal material would exhibit comparatively long *t*_w_ and short *t*_s_, both on the scale of minutes, which would allow for both confident operation by the dentist and short waiting time for both dentist and patient. However, the two time response requirements counteract to each other and obviously a trade-off must be accepted.

The characteristic times of impression materials have been measured traditionally by shark fin test (SFT) apparatus [4] and hand portable durometers based on Shore hardness scale [5]. These simple techniques are inexpensive, easy to use, and allow for a fast measurement in real time during the setting, making it possible to directly access the material kinetics. More accurate physical techniques that allow for elastic measurements, for example nanoindentation—via either traditional indenters [6] of atomic force microscopy [7]—or bending in dynamic mechanical analysis [8,9,10] or compression/tension in universal testing machine [1,11,12], are more complex and require 10–30 min for each measurement, due to specimen preparation and loading. On the other hand, durometer and SFT measurements suffer from a poor scientific background. The only advanced instruments usable so far for both accurate and fast analysis of impression materials are viscosimeters or rheometers [13]. However, alternative means with similar performance and non-invasive character may also be found in Raman or infra-red (IR) spectroscopy, which today are routine techniques for the investigation of polymers through their molecular vibrations [14]. IR in particular has found widespread application thanks to its convenient implementation as Fourier-transformed IR (FTIR) [15].

In this work, in addition to SFT and durometer we also used FTIR to study the time response of dental impression materials for the first time to our knowledge. Application of advanced nanoscience and nanotechnology practices in dentistry is an expanding reality [16,17,18], for example the measurement of surface roughness and stiffness via atomic force microscopy has become a new standard technique with unsurpassed sensitivity and resolution [19,20]. In this streamline, FTIR should make it possible to explore the connections between composition and functionality of impression materials.

The null hypothesis which we aimed to check here by means of the experimental techniques described above is that the working times specified by the manufacturers are in agreement with the results of instrumental tests done in the laboratory.

## 2. Results

**Figure 1:**
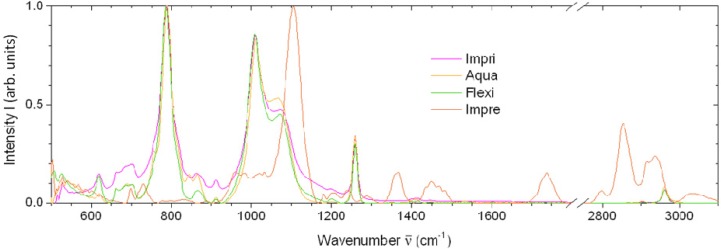
Fourier-transformed IR (FTIR) spectra of all the four materials investigated, at the shortest possible acquisition time since mixing (t ≈ 15 s).

**Figure 2:**
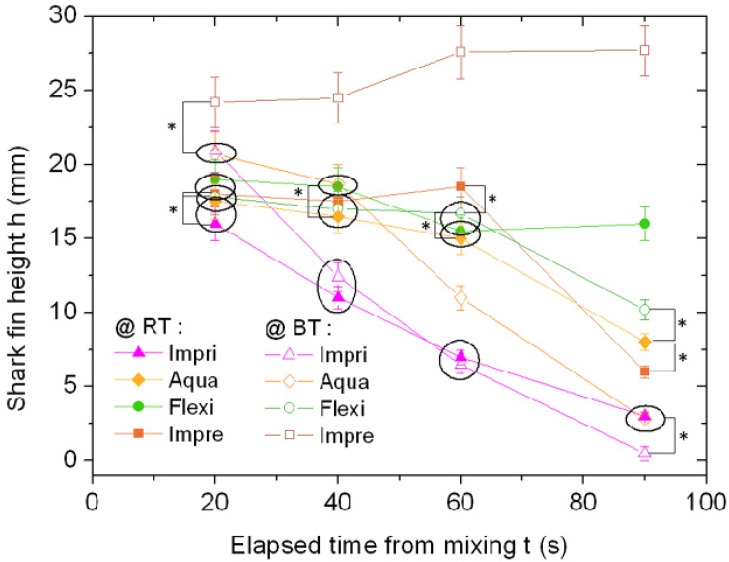
Shark fin test (SFT) measurements for the four materials made at room temperature (RT) (filled symbols) and body temperature (BT) (void symbols).

**Figure 3:**
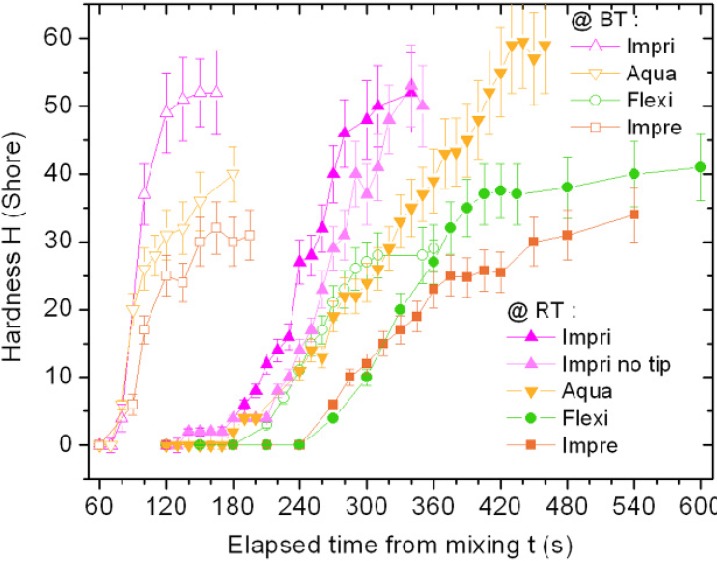
Shore hardness measurements for the four materials made at RT (filled symbols) and BT (void symbols).

In Figure 1, Figure 2 and Figure 3, the colors of the data points for the different materials have been assigned to be as close as possible to the actual material colors. In Figure 1 one representative set of FTIR spectra for all the four materials at the shortest possible time from mixing (~15 s) is shown, across the wavenumber range where major features emerge. Clearly, the spectra of all three ASs are similar, whereas the spectrum of the PE material is quite different. Thus, FTIR first allows us to group the materials into classes with similar chemistry.

Next, we try to assign some prominent features in Figure 1. The Impri spectrum (magenta line) shows the strongest peaks at ~789, ~1011 and ~1259 cm^−1^. The first is assigned to the Si–C bond, and was used as the reference peak for normalization. The peak at ~1011 cm^−1^ is assigned to the Si–O bond. On the long wavenumber side of the 1011 cm^−1^ peak a shoulder due to a secondary peak at around 1068 cm^−1^ also appears, which is assigned to Si–O–Si asymmetric stretching. In fact, the whole 1000–1100 cm^−1^ broad band is distinctive of siloxane Si–O–Si bonds [21]. The peak at 1259 cm^−1^ can be assigned to a Si–CH_3_ symmetric bending [21]. Additional features of Impri spectrum arise probably due to silica nanoparticle fillers, which are typically used for fine tuning of either rheological or stiffness properties of the fluid and set material, respectively.

For the other AS materials only minor differences appear with respect to Impri in the fingerprint region. For example, in Aqua a shoulder appears at ~758 cm^−1^, tentatively assigned to C–O [22] or, together with bands at 708, 716, 728 and 758 cm^−1^, to the CO_3_^2−^ in-plane bending [23]. Possible assignment to a styrene component can be excluded, as that should also appear in another sharp peak at 704 cm^−1^ which is absent [24]. Additionally, the 912 cm^−1^ peak splits into two peaks at 843 and 865 cm^−1^. The spectrum of Flexi also looks similar to both above, but closer to Impri, with no above peak shoulder and splitting.

For the spectrum of Impre, the highest peak appears at ~1105 cm^−1^, which is the C–O stretching typical of ether C–O–C linkage in the 1000–1300 cm^−1^ range [21]. The second highest peak is at 2853 cm^−1^, due to the CH stretch. Peaks at 698 and 730 cm^−1^ could be due to =CH bending. The band around 995 cm^−1^ could be the crystalline band due to symmetric stretching of C–O–C [25]. The bands at 2939 and 2922 cm^−1^ could be due to methylene (CH_2_) stretching [25]. Multiple peaks also appear within the 1250–900 cm^−1^ band, which could be due to cyclic rather than aromatic ether [21]. The peaks at 1364, 1738, 2795 and 2930 cm^−1^ are compatible with those observed in [25]. Finally, the bands at 1460 and 3030 cm^−1^ could be CH_2_ scissoring and =C–H stretching [21].

The difficulty in assigning all the peaks is due to the presence of additional components mixed in our materials, including nanoparticles, color pigments, and possible retarders or accelerators to appropriately tune the time response. However, identification of those peaks that change intensity over the setting time scale is of major interest here, as discussed at the end of this section.

Characterization of the temporal response of the impression materials is straightforward when using the SFT and Shore hardness techniques. In Figure 2 the SFT heights taken for the four materials at different times are shown. Each datapoint represents the mean of several measurements (*N* ≥ 4). The standard error was approximately 7% for most datapoints, and it has been represented in Figure 2 as error bars corresponding to ±1 σ (standard deviation). In Figure 2 also the most significant results of analysis of variance (ANOVA) with Bonferroni pair comparison tests have been represented: the datapoints enclosed by circles were not significantly different among each other (*p* > 0.05), whereas for pairs of datapoints statistically different (*p* < 0.05) a line connecting the points and signed by a single star (*) is displayed. All the other pairs without either enclosing circle (no difference) or line with star (difference at 0.05 level) must be considered being even more significantly different (*p* < 0.01), which would require a double star (**) as the graphic description, not placed on the graphic for the sake of clarity. The measurements have been repeated with device at room temperature (RT) and at body temperature (BT) at the time of pouring the mixed material into the mold. At BT (void symbols in Figure 2) the three ASs have similar behavior, since the shark fin height starts at the shortest time (*t* = 20 s) from similar values (18–21 mm) and decreases in all cases, even if in a milder way for Flexi and faster than all for Impri. On the contrary, Impre shows an anomalous behavior, since the fin height does not decrease with time in the considered range. At RT (filled symbols in Figure 2) instead, the Impre fin height decreases with time showing similar trend to Impri and Aqua (the former still being the fastest one in decrease, even if less fast than for BT). On the contrary, Flexi stays quite constant in fin height during the considered time, similar to Impre at BT. Thus, Flexi at RT does not crosslink in one minute, whereas it starts to change at BT: this probably occurs due to a special component, described by the manufacturer as “advance termasense” formulation [26]. Obviously, an increase in temperature activates an increase of viscosity for Flexi and slows down the increase of viscosity for Impre. Additionally, Impre values at BT are shifted to higher values than for the corresponding datapoints of the same material at RT, at all times, and also *versus* all the other materials, which means a significant decrease in viscosity for Impre with the increase in temperature.

The above datapoints in Figure 2 have been used to extract *t*_w_ values, taken as the intermediate time at which the shark fin height has decreased to half its initial value. It is assumed here that the SFT height correlates mostly with the fluidity of the material before setting. The results of this analysis are listed in Table 1.

**Table 1:** For each cell, the top row value is *t*_w_ and the bottom row one is *t*_s_, where available. All numerical values are means (*N* ≥ 4) in seconds. The column of dentist’s perception is a qualitative score, higher score means best, + shows only a slight preference. §: for all “heavy body” formulations.

Materials	Methods of Assessment							
	Manufacturer Specifications (BT)	Dentists’ Perception (BT)	Durometer			SFT		FTIR (RT)
			Literature [27] §	RT	BT	RT	BT	
Impri	60	1	-	-	-	55	46	68
(light‑bodied)	95	-	225	223	98	-	-	353
Aqua	70	2	-	-	-	87	62	57
(regular set)	300	-	300	319	115	-	-	284
Flexi	180	2+	-	-	-	>>90	>90	>600
(medium flow)	360	-	350	332	251	-	-	>>600
Impre	60	3	-	-	-	83	>>90	>41
(light‑bodied)	240	-	-	330	117	-	-	>185

Similarly, we assumed that the Shore hardness increasing over the time (see Figure 3) is instead a measurement of the solidification of the material, *i.e.*, its setting. Therefore, in that case the time up to one half of the final hardness has been used to obtain *t*_s_ values (see Table 1).

Qualitatively, the hardness *versus* time profiles in Figure 3 are much more finely sampled than the SFT ones, since the respective measurements are faster and could be made all on the same specimen, in just another small region close to the previous ones. Similar to Figure 2, also in Figure 3 the error bars represents ±1 σ (the standard error being here approximately 12%). However, given the large number of datapoints for these measurements, only the most significant values (*i.e.*, time at half of the maximum final hardness and final hardness) have been compared with ANOVA (results not shown in the plot, for the sake of clarity). The hardness curves at BT (void symbols) have similar intermediate time (*p* > 0.05) and are all shifted towards shorter times with respect to the corresponding curves at RT (*p* < 0.01) but for Flexi, which at BT shows similar *t*_s_ (*p* > 0.05) than the other three materials at RT. Thus, heat accelerates material setting, and only Flexi seems relatively insensitive to this effect, as its BT curve is also shifted to shorter times than the respective RT curve (*p* < 0.01), yet to a much lower extent (approximately −20% only in *t* instead of the −65% for the other materials). For Impri, an additional comparison between spiral tip and manual mixing points out that in the latter case setting is only slightly slower (*p* < 0.05), likely due to less uniform mixing.

The final hardness values of Impri are similar between BT and RT datasets (*p* > 0.05), while Aqua at RT reached a much higher value than at BT (*p* < 0.01) and higher than all other materials at both temperatures (mostly *p* < 0.01, only *p* < 0.05 for Impri at both RT and BT): thus, heating made Aqua to set slower yet to higher extent. The maximum final hardness at RT was higher than that at BT also for Flexi (*p* < 0.05), whereas it was not for Impre and Impri (*p* > 0.05).

**Figure 4:**
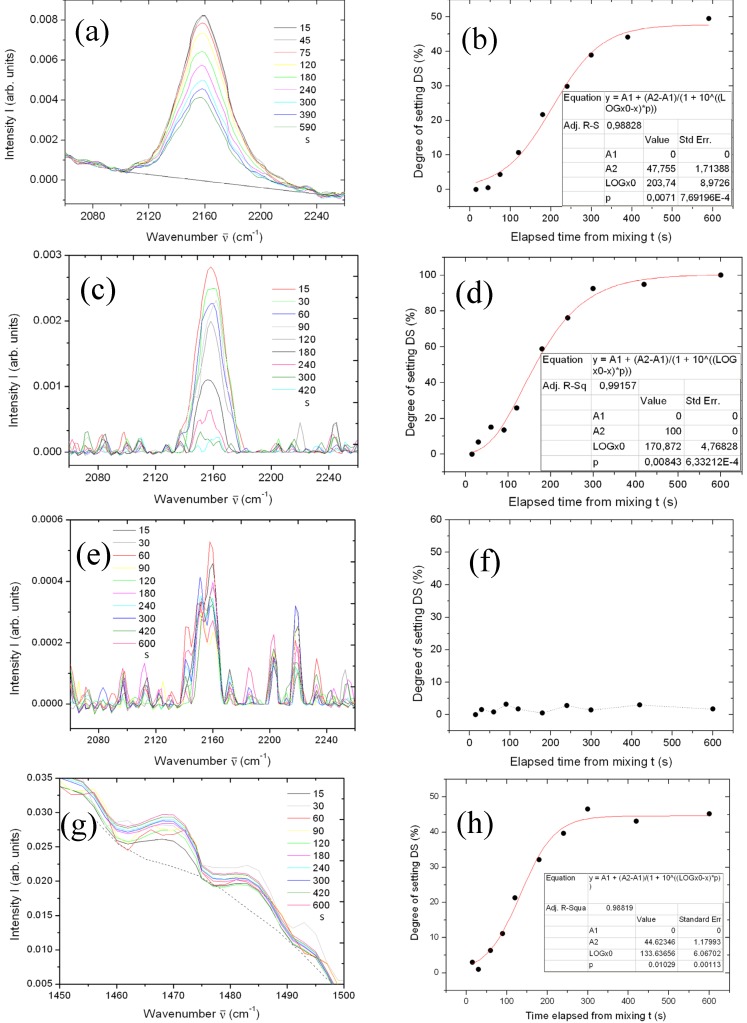
(**a**), (**c**), (**e**) and (**g**): regions with the peaks selected as representative of the expected setting process of Impri, Aqua, Flexi, and Impre over the time. (**b**), (**d**), (**f**) and (**h**): DS calculated from the respective peak signal, *versus* time, and—where possible—sigmoidal fit to the change.

Finally, from the FTIR spectra of the four materials as in Figure 1 but at different times since mixing, we sought the peaks whose intensities were changing over the time, to describe the materials setting. Candidate peaks are plotted in the left column of Figure 4 (Figure 4a,c,e,g). From the change in peak intensity, where available, we calculated discrete degree of setting (DS) values (right column of Figure 4, *i.e.*, Figure 4b,d,f,h) and fitted these datapoints *versus* time with a sigmoid. This curve has been used to find the times at which DS increased up to 10% and 90% of its final maximum value, defined by us as the *t*_w_ and *t*_s_, respectively. Thus, we assumed that the FTIR information described the full materials physics and chemistry, and used its initial deviation from the starting value (+10% in units of final maximum) and its approach to the final value (−10% with respect to maximum) as markers for the respective characteristic times. Again, the numerical results appear in Table 1. Deviations between the significant peaks of different spectra (*N* ≥ 2, details see Material and methods) were all below 10%, so similar absolute errors (±10%) are expected in the resulting *t*_w_ and *t_s_* values obtained with this method.

## 3. Discussion

To our knowledge, FTIR has been used so far on dental impression materials only to assess the amount of OH groups present on the surface and characterize the wetting properties [28]. Here a new use is attempted to assess the time response during setting. With respect to the SFT and Shore hardness datasets, the FTIR ones—where available—are more detailed in the transition (*i.e.*, a better fit can be made), and account for the chemical changes at molecular level which comprise both the viscous and elastic properties of the material, so can provide both *t*_w_ and *t*_s_.

FTIR is commonly used to assess the setting of dental restorative resins, based on the chain network change in double C bond during polymerization [8]. The chemistry of dental impression materials is obviously different. As the Si chain in ASs replaces the C chain in resins, bonds to Si have to be considered to evaluate the ASs reticulation. In the simplest picture, we expect the change in viscosity assessing the working time to be described by the same setting reaction, despite the complexity of reactions engineered in the materials formulation with the goal of tuning *t*_w_ and *t*_s_ to opposite requirements.

Since Si does not form a stable chain with itself [1], stability is obtained in silicones by interpolation of O, forming polysiloxanes. The pending Si bonds along the chain can be saturated by H, but the Si–H bond is extremely reactive and is usually substituted by an alkyl group like methyl (CH_3_), making the Si–C bond inert. If such a chain is terminated with hydroxyl groups (OH) the molecule is called a silanol, and this is called a condensation silicone, after the polymerization reaction used for its manufacturing [1] via elimination of small molecules like H_2_O. The dental impression materials are usually ASs, thanks to their better biocompatibility and higher stability, given by a variety of factors preventing them from curing. In ASs chain termination group is a vinyl (–CH=CH_2_), so one has poly-vinyl-siloxane or vinyl-poly-siloxane (VPS), a moderately low molecular weight silicone that contains silane groups [2].

The main IR absorptions of silicones, appearing at ~770, 1000–1100 and ~1260 cm^−1^, allow for detection down to 1% level sensitivity [29]. For the time progression of silicones setting one peak of Si–O should be found to be increasing, concurrently with a decreasing Si–H peak. Indeed, for Impri and Aqua we found a significant decrease of the Si–H peak at 2158 cm^−1^ over time, and used this peak according to Equation (1). Unfortunately, for Flexi the 2158 cm^−1^ peak was less clearly defined and did not change with time (see Figure 4e,f). Additionally, we failed to identify other FTIR peaks that exhibited a clear trend in time. One possible explanation (see Table 1) would be that the characteristic times of change are much longer than those considered here. Another possibility is that the reaction chemistry of Flexi is not associated with FTIR-active vibrations, and alternative spectroscopic techniques such as Raman scattering should be used. We rather think that for Flexi the setting process in more complex than for Impri and Aqua and can not be described by a single peak change, but a chain of reactions is involved which is hard to reconstruct without detailed information from the manufacturer.

For Impre a single peak clearly changing with time (increasing) was identified at 3032 cm^−1^ (data not shown). However, when assessment of DS was attempted according to Equation (2), the obtained datapoints provide values of 36 and 184 s for *t*_w_ and *t*_s_, respectively, quite far from the SFT and durometer tests. We speculate that the 3032 cm^−1^ peak is due to an initiation reaction. In fact, it is expected that the aziridine ring opening is promoted by the action of an aromatic sulfonate ester [30]. Alternatively, after the assumption that PE is cured via cationic polymerization of aziridine rings [31], we tried to use for Impre the peak at 1240 cm^−1^ (data not shown). In this case the ring opening should be accompanied by decrease in C–N amine bonds stretching signal, to appear in the 1080–1340 cm^−1^ range. However, the 1240 cm^−1^ peak showed no change up to the times considered (10 min at RT). Instead, similar to the observation in [32] for PEEK, a weak band at 1470 cm^−1^ was observed to increase, which could be due to sulfonation starting the crosslinking (see Figure 4g). This band and that one at 1483 cm^−1^ were tentatively used as the signal and reference, according to Equation (2), to obtain the DS datapoint in Figure 4h. From the resulting best fit curve, again very short time values as for the 3032 cm^−1^ peak were found, namely 41 and 185 s. We assume that these values are just a lower limit for characteristic times, related to the initiation only of the PE crosslinking via sulfonation [1].

In Table 1 also the qualitative opinions of the two dentists participating in this study have been included for the working time, which is the parameter of highest practical interest. According to the scores describing their clinical experience, they agree that the best material in workability is Impre, followed by Aqua and Flexi with a slight preference only for one dentist for the latter. This is confirmed by the laboratory tests presented here, even if in contrast with the manufacturer specifications, which show a *t*_w_ for Impre as short as for the other three ASs.

In commenting the numerical data summarized in Table 1, one can first observe that, as expected, the intermediate time obtained by the SFT trends (representing *t*_w_) is significantly lower than that obtained by the durometer measurements (representing *t*_s_). Obviously, the former measurement is related to the properties of the material as a viscous paste, whereas the second is related to the properties of the material as a solid, after setting into the final mold.

With respect to the nominal values from the manufacturers, concerning the *t*_w_ obtained from SFT: Impri, Aqua, and Flexi are in good agreement; Impre instead shows longer *t*_w_, even at BT (where it is actually even longer than at RT). Concerning the *t*_s_ obtained from the durometer: Impri is in agreement with the nominal value at BT (but is much longer at RT, in agreement with the literature); Aqua is in agreement with the nominal value at RT (but is much shorter at BT); Flexi is in agreement at RT (but shorter at BT); Impre has RT and BT *t*_s_ around the nominal value (but the RT one is closer).

Concerning the FTIR results, for Aqua both *t*_w_ and *t*_s_ are in agreement with the other techniques and the nominal values, whereas for Impri the *t*_w_ is in agreement with the nominal value but the *t*_s_ seem to be much longer.

## 4. Experimental Section

The materials were selected and provided by the dentists involved in the study. They were three materials from the AS class, namely Imprint 4 (3M-ESPE, St. Paul, MN, USA) [27], Flexitime (Heraeus Kulzer, Hanau, Germany) [26] and Aquasil (Dentsply, Konstanz, Germany) [33], and one from the PE class, namely Impregum (3M-ESPE) [34]. In the following they have been named shortly Impri, Flexi, Aqua and Impre, respectively. The materials were dispensed with a gun for dual-compartment cartridges equipped with ~7 cm long tips with spiral-shaped baffle, providing automatic mixing of the base and catalyst in the required volume proportions (1:2 for Impre and 1:1 for all the other materials).

FTIR was performed on a Vertex 70 (Bruker Optics, Billerica, MA, USA) in attenuated total reflection. The spectra were acquired on the whole range of 400–4000 cm^−1^, with an aperture size of 3 mm and a resolution of 4 cm^−1^. For minimization of noise effects, five scans were averaged per spectrum and apodized by Blackman-Harris three-term correction function. After acquisition of the transmission signal, the spectra were converted into absorbance in arbitrary units and the baseline was calculated with 3rd order polynomial and removed. Finally, the spectra were normalized to the highest peak intensity. The number of measured spectra at each time was *N* = 2 for Impri and Aqua, *N* = 3 for Impre and *N* = 4 for Flexi.

For SFT measurements, both an original SFT device of stainless steel by 3M-ESPE (lent on purpose) and a home-made SFT device of aluminum were used, which demonstrated equivalent results within the experimental error. Before each measurement the SFT device was either kept at room temperature (RT, 22 ± 2 °C) or heated at body temperature (BT, 37 ± 1 °C) for 10 min in a thermostated water bath. In the latter case, on removal from the bath the device was quickly dried by wiping with cotton cloth and blowing with compressed air (total time ~10 s). The device was loaded with ~6 mL of mixed material, in ~5 s, and this time was summed up with extra rest time, before letting the SFT piston equipped with V-shaped slit gently touch the polymer surface and start sinking under ~147 g load. The slit had 1 and 2 mm arch length for the tests made at RT and BT, respectively. The total time before sinking start was taken as the independent variable for the measured shark fin height. Time values of 20, 40, 60 and 90 s were used for all the materials. After 10 min the SFT device was disassembled, the excess material flown out of the split ring was cut off, and the shark fin height was measured with a caliper. Whereas the instrumental error was ±0.02 mm, a measuring accuracy of ±0.1 mm was assumed due to operator uncertainty in assignment of fin limits. All measurements were repeated at least four times (*N* ≥ 4).

The durometer measurements were taken with a Affri 3001 Shore A portable hardness tester (BAMR, Cape Town, South Africa). The sensing tip of this device protrudes from the reference end-of-scale head level of ~1.5 mm. A metal mold was used for the material to be indented, of ~7 cm length, ~0.5 cm width, and ~4 mm depth. The mold depth was high enough that, on full penetration in the material, the durometer tip was still far from touching the mold bottom. The mold width was small enough that the durometer head was sitting on the top sides of the mold as a reference level. The mold length was high enough for each measurement to be taken at a position 2–3 mm apart along the specimen, not to be affected by the previous one. The 100 end-limit of A Shore hardness scale, according to manufacturer’s calibration, corresponds to ~3.8 kg load. Assuming a spherical tip apex of ~0.5 mm diameter, this load would mean a maximum stress of ~190 MPa. The measurements were repeated at 10 s intervals after the first 1 min since mold loading, to avoid the initial full sinking in the liquid material, which would spoil the tip and affect the subsequent measurements. Same as for SFT, the materials were always coming from storage in the ambient, but the metal mold was either at RT as well or heated up to BT by means of a hotplate. All measurements were repeated four times (*N* = 4).

To try to extract both *t*_w_ and *t*_s_ from the FTIR spectra, a quantity called degree of setting (DS) has been defined as follows. Assuming that a peak decreasing with time due to setting is identified, its intensity *I*_sign_—taken as the signal—is normalized to the intensity of a reference peak from the same spectrum, *I*_ref_, and the ratio *I*_sign_/*I*_ref_ is rescaled between each time instant *t* and the respective constant value from the initial spectrum at time *t*_0_:

DS = 100[1 − (*I*_sign_/*I*_ref_)(*t*)/(*I*_sign_/*I*_ref_)(*t*_0_)] (1)

Equation (1) is similar to that used for resin composites to assess the degree of conversion resulting from polymerization, where *I*_sign_ is usually the intensity of the peak at 1637 cm^−1^ and *I*_ref_ that at 1580 cm^−1^, related to stretching of the aliphatic and aromatic C=C bonds, respectively [8]. If, on the contrary, the peak used for *I*_sign_ is increasing over the time, the following formula should be used to have a DS increasing with time:

DS = 100[−1 + (*I*_sign_/*I*_ref_)(*t*)/(*I*_sign_/*I*_ref_)(*t*_0_)] (2)

In addition to the above quantitative evaluations of the characteristic times of the materials, we also asked the two dentists involved in the study to provide a qualitative evaluation of the material performances, limited to the working time. This opinion was based on a number of clinical treatments *N* ≥ 10 for each considered material, in several different conditions of treated patients.

## 5. Conclusions

As a method for assessing the working time of dental impression materials, the SFT alone is commonly used. Yet, this empirical technique does not give insight in the physics and chemistry of setting and has long being criticized in the literature. Complementary techniques based on stronger scientific foundations should also be used for this task. We have studied the behavior of four dental impression materials based not only on SFT and durometer but also on FTIR measurements, probing the molecular bond vibrations. The materials investigated were one PE (Impre) and three ASs (Impri, Flexi, Aqua), which were studied *versus* the time since mixing at intervals as short as 15–20 s. This allowed us to extract the characteristic working and setting time of the polymers, after appropriate definition. The null hypothesis that laboratory measurements would agree with the nominal time values from the manufacturers was not confirmed. There exists an overall ambiguity on the temperature of supposed assessment, if BT or RT, whose experimental results are closest to the nominal values. Additionally, the working time of Impre turned out to be longer than the nominal value. For Flexi and Impre no single time-evolving peaks associated with their setting reaction could be identified from FTIR. This may be a limitation intrinsic in the technique, which in the future should be complemented with additional information on the material formulations or with techniques such as thermogravimetric analysis or differential scanning calorimetry.
